# Accuracy of endoscopic ultrasound-guided fine-needle aspiration in the suspicion of pancreatic metastases

**DOI:** 10.1186/1471-230X-13-63

**Published:** 2013-04-11

**Authors:** José Celso Ardengh, César Vivian Lopes, Rafael Kemp, Filadélfio Venco, Eder Rios de Lima-Filho, José Sebastião dos Santos

**Affiliations:** 1 Endoscopy Unit, Hospital 9 de Julho, São Paulo, Brazil; 2 Division of Surgery and Anatomy, Ribeirão Preto Medical School – University of São Paulo, São Paulo, Brazil; 3 Endoscopy Unit, Santa Casa Hospital, Porto Alegre, Brazil; 4 Endoscopy Unit, Moinhos de Vento Hospital, Porto Alegre, Brazil; 5 Diagnostika Pathology Unit, São Paulo, Brazil; 6 General Surgery, Hospital Federal dos Servidores do Estado do Rio de Janeiro, Rio de Janeiro, Brazil

**Keywords:** Pancreatic neoplasms, Metastasis, Endosonography, Biopsy, Fine-needle, Histology

## Abstract

**Background:**

Metastases to the pancreas are rare, and usually mistaken for primary pancreatic cancers. This study aimed to describe the histology results of solid pancreatic tumours obtained by endoscopic ultrasound-guided fine-needle aspiration (EUS-FNA) for diagnosis of metastases to the pancreas.

**Methods:**

In a retrospective review, patients with pancreatic solid tumours and history of previous extrapancreatic cancer underwent EUS-FNA from January/1997 to December/2010. Most patients were followed-up until death and some of them were still alive at the end of the study. The performance of EUS-FNA for diagnosis of pancreatic metastases was analyzed. Symptoms, time frame between primary tumour diagnosis and the finding of metastases, and survival after diagnosis were also analyzed.

**Results:**

37 patients underwent EUS-FNA for probable pancreas metastases. Most cases (65%) presented with symptoms, especially upper abdominal pain (46%). Median time between detection of the first tumour and the finding of pancreatic metastases was 36 months. Metastases were confirmed in 32 (1.6%) cases, 30 of them by EUS-FNA, and 2 by surgery. Other 5 cases were non-metastatic. Most metastases were from lymphoma, colon, lung, and kidney. Twelve (32%) patients were submitted to surgery. Median survival after diagnosis of pancreatic metastases was 9 months, with no difference of survival between surgical and non-surgical cases. Sensitivity, specificity, positive and negative predictive values, and accuracy of EUS-FNA with histology analysis of the specimens for diagnosis of pancreatic metastases were, respectively, 93.8%, 60%, 93.8%, 60% and 89%.

**Conclusion:**

EUS-FNA with histology of the specimens is a sensitive and accurate method for definitive diagnosis of metastatic disease in patients with a previous history of extrapancreatic malignancies.

## Background

Pancreatic metastases (PM) are rare [1], accounting for 3% of pancreatic solid tumours submitted to surgical resection [2]. PM can be identified during staging of a disseminated cancer or during periodic control of a primary cancer treated several months or years before [3,4]. More rarely, PM can be found even before the detection of the primary tumour, a situation in which the poor prognosis and low rate of resectability are well known [5-7]. The misdiagnosis of a primary pancreatic cancer can be avoided by performing a preoperative biopsy, either through a percutaneous radiologic method (US or CT) or by endoscopic ultrasound-guided fine-needle aspiration (EUS-FNA) [8].

EUS-FNA is the least invasive and most effective method for diagnosis of pancreatic tumours [8,9]. However, its importance for diagnosis of PM has been reported only by description of isolated cases [6,7,10-20] or small case series [3,4,21-23] describing and comparing PM endosonographic imaging and cytological findings with primary pancreatic cancer [3]. There is no large case series with history of previous extra-pancreatic malignancy followed-up until death in which the diagnosis of PM has been made through the histology obtained by EUS-FNA.

The aim of this study was to determine the incidence, clinical characteristics, endosonographic patterns, survival, and performance of the EUS-FNA in a large case series suspicious for PM.

## Methods

### Patients

Between January 1997 and December 2010, 1986 patients with radiological evidence for a solid pancreatic tumour or enlargement of the pancreatic head underwent EUS-FNA at Endoscopic Ultrasound Units from Hospital 9 de Julho and Hospital das Clínicas / Ribeirão Preto Medical School. This retrospective study was based on the microhistologic analysis of pancreatic metastases obtained by EUS-FNA from patients with or without a previous history of extrapancreatic cancer. Those whose pathology examination obtained at the time of surgery or EUS-FNA had no definitive diagnosis of a pancreatic neoplasia (atypia, suspicion of malignancy, and insufficient material for immunohistochemistry), patients with a pancreatic mass, history of previous extrapancreatic cancer and FNA confirming a primary pancreatic cancer, as well as those with a primary pancreatic lymphoma were excluded. Ethical approval for this retrospective study was obtained from both institutional review boards.

Demographics, clinical features and endosonographic findings were recorded. The information about every case was obtained through e-mail and/or phone call to the referring physicians and family members, and by review of the medical charts. Only the radiologist and pathologist were blinded to the history of previous cancer for all the patients in this study.

### EUS-FNA

Once informed consent was obtained for the procedure, patients were sedated with propofol associated with midazolam and fentanyl under cardiorespiratory monitoring. All procedures were performed by the same echoendoscopist with a large experience in diagnostic and therapeutic echoendoscopy (JCA). The sectorial echoendoscopes used were: Pentax FG 38-UX (Pentax Precision Instruments Corp., Orangeburg, New York) coupled to an ultrasound unit EUB 515 (Mitsubishi, Conshockon, Philadelphia), Olympus UCT-160 OL5 (Olympus Optical Corp., Ltd., Tokyo, Japan) coupled to an ultrasound unit UC-60 (Suzy-Olympus Optical Corp. Ltd., Tokyo, Japan), and Fujinon EG-530UT (Fujifilm Optics Corp. Ltd., Sano, Japan) coupled to an ultrasound unit SU7000 (Kodai Hi Tec Corp. Ltd., Saitama, Japan). Only needles of 22 gauge and length of 145 cm (Medi Globe, Medizintechnik GMBH, Grassau/Germany) were used for all the punctures. The endosonographic features taken into account were: location, size, echotexture, homogeneity, borders and proximity to blood vessels. After puncturing the tumour, core specimens were obtained by flushing the needle with 2 ml of saline and then by reintroduction of the stylet inside the needle. All material was placed in 10% buffered neutral formalin solution. As an on-site cytopathologist was not available in our routine, the specimens were considered satisfactory in the presence of non-hemorrhagic small tissue filaments or tissue core samples. The specimens were sent to a pathologist (FV), and prepared according to a previously described cell block technique [24].

### Statistical analysis

Statistical analysis of continuous variables was described as mean and standard deviation, and dichotomous variables were expressed as simple ratios. The sensitivity, specificity, positive and negative predictive values, and accuracy of the histology findings obtained by EUS-FNA for the diagnosis of PM were calculated, as well as their 95% confidence intervals. Sensitivity was used to evaluate the capacity of the EUS-FNA to detect the patients that were known to have pancreatic metastases. Specificity was used to evaluate the capacity of the EUS-FNA to confirm the cases that were known not to have pancreatic metastases. The positive predictive value analysed how likely patients with pancreatic metastases detected by EUS-FNA actually did have the finding, and the negative predictive value analysed how likely patients without detection of pancreatic metastases by EUS-FNA actually did not have the finding.

## Results

### Patients

Fifty-two patients (2.6%) were identified. After applying exclusion criteria for suspicious lesions for PM, 37 patients were selected. Their demographics are presented in the Table 1. All patients were evaluated by computed tomography before the EUS-FNA, which detected a pancreatic mass in 32 (85%) cases, an increase in size of the pancreatic head with jaundice but no focal mass in 2 (6%) cases, a normal pancreas in another two (6%) cases (one of them with jaundice), and a segmental dilation of the main pancreatic duct in 1 (3%) patient. One case mistaken by CT as a gastric subepithelial tumour with normal pancreas was revealed by EUS as a pancreatic mass.

**Table 1:** Demographic findings of the patients

**Characteristics**	**n (%)**
**Age (average)**	60.3y (26–84 y)
**Gender (male/female)**	26 (70) / 11 (30)
**Symptoms**	24 (65)
Abdominal pain	11
Abdominal pain + weight loss	6
Jaundice	5
Acute pancreatitis	2
**Asymptomatic**	13 (35)
**Diagnosis of metastases**	
**Control of disease**	29 (78)
Non-Hodgkin lymphoma	6
Colon cancer (adenocarcinoma)	4
Renal cancer (clear renal cell cancer)	4
Breast cancer	3
Sarcoma [Leyomiossarcome (1), Rhabdomiossarcome (1) and sarcoma (1)]	3
Gastric cancer (adenocarcinoma)	2
Skin cancer (melanoma)	2
Bladder cancer	1
Esophageal cancer (squamous cell carcinoma)	1
Gallbladder cancer	1
Lung cancer (NSCLG)	1
Mieloma Multiplus (Plasmocytoma)	1
**Before identification of the primary cancer**	6 (15)
Renal cancer (clear renal cell cancer)	1
Lung [SCLC(1) and squamous cell carcinoma (2)]	3
Ovarian cancer	1
Mesothelyoma	1
**Initial Staging**	2 (6)
Gastric cancer (signet ring cells adenocarcinoma)	1
Liver (hepatocellular carcinoma)	1

*NSCLC*: non-small cell lung cancer; *SCLG*: small cell lung cancer.

Control of disease: periodical control of a previously identified extrapancreatic tumour.

Before identification of the primary cancer: Imaging methods revealed a pancreatic mass, and EUS-FNA confirmed it as a metastatic tumour, without detection of the primary cancer.

Initial staging: an extrapancreatic tumour and a pancreatic mass or enlargement were detected at the same time.

### EUS evaluation

**Figure 1:**
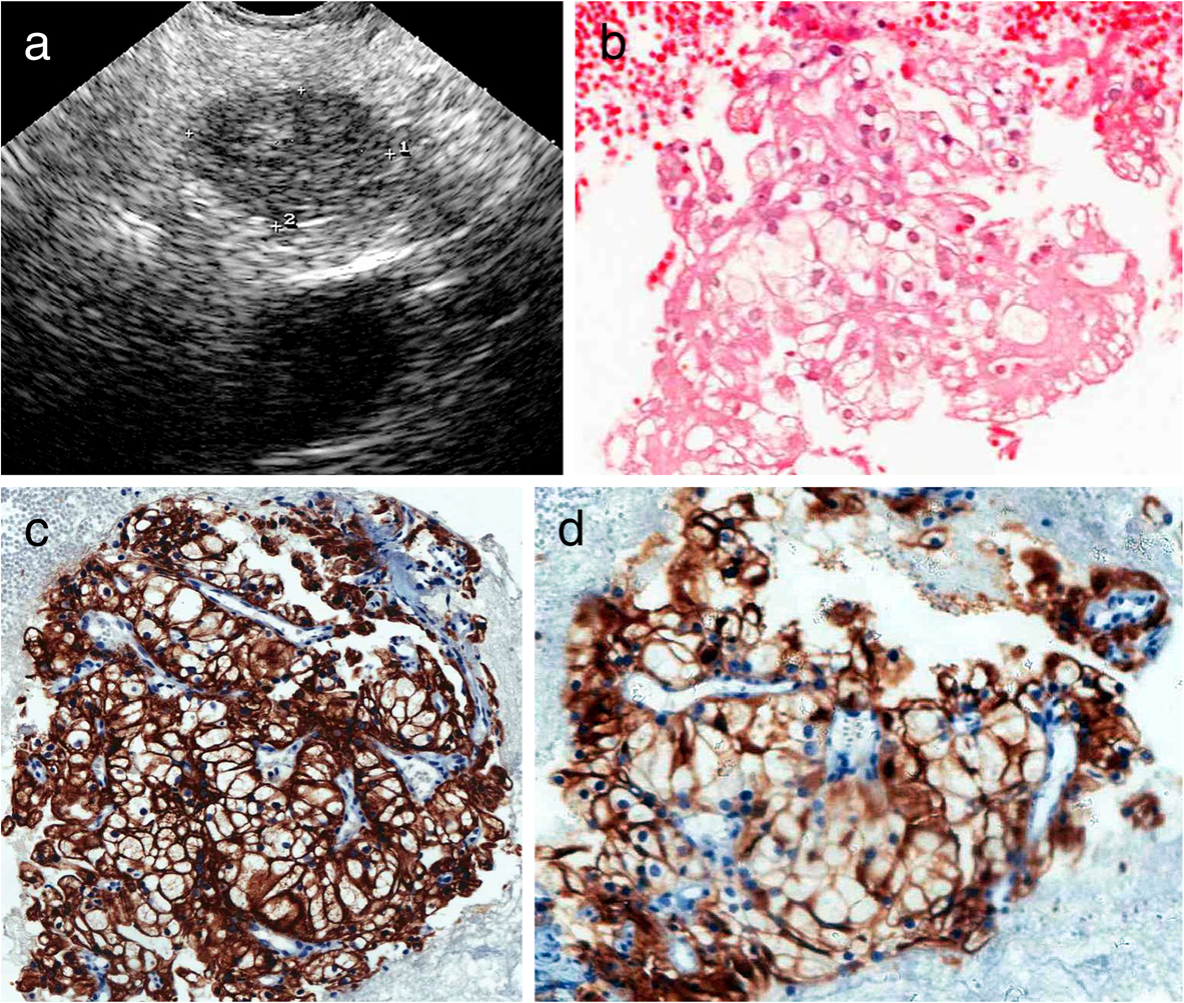
**Asymptomatic patient (n° 37) submitted to surgery due to clear renal cell cancer 30 years ago.** (**a**) EUS revealed a hypoechoic, homogeneous, with well-defined borders lesion in the pancreatic body. (**b**) Histology obtained by EUS-FNA of the lesion was suggestive of a renall cancer. (**c**) Imunohistochemistry with positive reaction for acidic cytokeratins (AE1) and (**d**) CD10 confirmed a metastasis from clear renal cell cancer.

The average size of the lesions was 42 ± 11 mm (range:12–127 mm). Lesions were located preferably in the head of the pancreas (21), but also in the body (9), tail (4), neck (1), body/tail (1) and head/body/tail (1). Most of the lesions were solid [32 (86%)], 4 were solid-cystic (esophageal cancer, renal cancer, mesothelioma, and tuberculosis) and one cystic (IPMN). In most cases lesions were solitary (34), hypoechoic (34), with well-defined borders (23) and heterogeneous (22) (Figure 1). Only 2 (5.4%) of them were hyperechoic (esophageal cancer and plasmocytoma) and another one was anechoic (IPMN). PM were hypervascular in 3 cases where Doppler signal was positive [renal (2) and hepatocellular carcinoma (1)].

EUS-FNA was successfully performed in all 37 patients after an average of 2.4 needle passes (range: 2–4). The final diagnosis was obtained in 35 (94%) cases: 30/35 confirmed the clinical suspicion for PM [lymphoma (6), colon (4), renal clear cell (4), lung (3), breast (2), leiomyosarcoma (2), stomach (1), esophageal (1), gallbladder (1), hepatocellular carcinoma (1), mesothelioma (1), myeloma (1), ovarian (1), rhabdomyosarcoma (1), and melanoma (1)]. A primary pancreatic neoplasm was confirmed in 3 cases [IPMN (1), NET (1) and adenocarcinoma (1)], and in 2 cases a pancreatic pseudotumour was found (pancreatic blastomycosis and tuberculosis). In the remaining 2 cases, the diagnosis obtained by EUS-FNA was chronic pancreatitis, but surgical resection confirmed a PM (gastric and lung cancer) (Additional file 1).

Twelve patients with pancreatic tumours were submitted to surgery. Four patients underwent exploratory laparotomy [colon cancer (2), gastric cancer (1) and NET (1)], 5 were submitted to gastroduodeno-pancreatectomy (colon, gastric, lung, renal clear cell and ductal adenocarcinoma) and 3 underwent subtotal pancreatectomy (renal clear cell (2), and lung cancer). The final diagnosis was colon cancer (3), renal clear cell (3), gastric cancer (2), lung cancer (2), ductal adenocarcinoma (1) and NET (1). Surgical diagnosis of PM was confirmed by histology obtained by EUS-FNA in 10 of 12 cases.

### Clinical impact of EUS-FNA

Metastases were confirmed in 32 cases. The incidence in this series was 1.6%. EUS-FNA confirmed the diagnosis of PM in 30/32 (94%) cases. For 29 cases with diagnosis of PM during cancer control, the mean and median time between diagnosis of the primary tumour and the PM were, respectively, 50.2 (2–360 m) and 36 months. For two patients in whom CT revealed nonspecific increase in the size of the pancreatic head, EUS-FNA made the diagnosis of lymphoma and PM from breast cancer. The first patient had been treated for non-Hodgkin's lymphoma for 6 months, and the other patient underwent surgery for breast cancer 5 years previously. The case in which CT showed only dilation of the main pancreatic duct, the patient had undergone surgery for a breast cancer 5 years earlier. Endosonographic features were suspicious for PM, but histology revealed a primary pancreatic adenocarcinoma, which was confirmed after surgical resection. In the patient presenting with jaundice and a normal CT, who had been treated for a non-Hodgkin's lymphoma for 6 months, EUS-FNA confirmed the diagnosis of PM from lymphoma. In the patient whose CT revealed a normal pancreas and showed a subepithelial gastric tumour, EUS-FNA detected a PM for ovarian cancer. In 3 patients in which EUS imaging was suspicious for a NET, EUS-FNA detected metastases [clear cell cancer (2) and hepatocellular carcinoma]. One patient with a pancreatic mass had had a nephrectomy for a renal cell tumour 30 years earlier and had a well-defined pancreatic nodule, which was resected. EUS-FNA made the diagnosis of PM prior to identification of the primary tumour in 6 (16%) patients [lung (3), kidney (1), mesothelioma (1) and ovarian cancer (1)]. Clinical features, treatment and follow-up of the remaining patients with clinical history of extrapancreatic cancer submitted to EUS-FNA are presented in the Additional file 1.

For calculations of sensitivity, specificity, positive and negative predictive values, and accuracy of EUS-FNA for diagnosis of PM, the patients with infectious pancreatic involvement mimicking a pancreatic mass, and the patient with IPMN were considered as true negatives; those in which EUS-FNA revealed chronic pancreatitis were considered as false negatives, and cases of NET and pancreatic adenocarcinoma, as false positives. The performance of EUS-FNA for diagnosis of PM are presented in the Table 2.

**Table 2:** Performance of EUS-FNA for diagnosis of pancreatic metastases

		**95% CI**
Sensitivity	93.8%	85% -100%
Specificity	60%	17% -100%
Positive predictive value	93.8%	85% -100%
Negative predictive value	60%	17% -100%
Accuracy	89%	79% - 99%

### Long-term follow-up after EUS-FNA

Most patients were followed-up until death. At the end of the study, only three of the patients with PM, 2 of them from renal clear cell cancer, and another one from breast cancer, were still alive, 6, 12 and 22 months, respectively, after EUS-FNA. The remaining 29 patients died, and the mean and median survival after diagnosis of PM were, respectively, 15.1 (range: 4–89 months) and 9 months. There was no significant difference in the median survival between patients who received surgery and those who did not (9 vs. 8.5 months).

## Discussion

Isolated pancreatic masses are usually primary pancreatic tumours, either ductal adenocarcinoma or neuroendocrine neoplasia, or represent focal chronic pancreatitis [3]. Secondary involvement of the pancreas by extrapancreatic cancer is rare and constitutes less than 3% of all pancreatic resections [2,4,25-27], though the rate varies between 3% and 12% of all pancreatic malignancies in autopsy studies [28]. Even though rare, the literature demonstrates the pancreas as a site for metastases from some cancers, especially renal, ovarian, breast, lung, brain and colon [2,4,6,7,23]. This data was corroborated by the findings of our series as well, with higher prevalence of non-Hodgkin's lymphoma, lung, renal, colon and breast cancer.

Patients presenting with pancreatic metastases often have non-specific symptoms. Patients can present with abdominal pain, weight loss, nausea, melaena, jaundice, and gastric outlet obstruction. Abdominal pain was the most frequent symptom in our patients, which was present in 46% of the patients. However, patients with pancreatic metastases can be asymptomatic in up to 35% of these cases [29]. This way, the symptomatology almost nothing contributes for diagnosis of a pancreatic metastases.

Some cases are encapsulated and hypervascular and may be similar to lymph nodes or pancreatic NETs [30,31], fact which occurred in 5 of our patients. Nevertheless, there is no pathognomonic imaging feature to make it possible to distinguish a PM from a primary pancreatic cancer [32-35]. This also occurred in our study, where CT did not suspect for a PM in any case. In our series, endosonographic characteristics of PM were similar to those found in primary pancreatic cancer in up to 50% of these cases, and small lesions tended to have well-defined borders, highly suggestive for pancreatic neuroendocrine tumours [3,4,21,22]. In the experience by DeWitt et al. [3], 46% of PM had well-defined borders when compared to 4% of primary pancreatic tumours (p<0.0001). Bechade et al. [36] demonstrated well-defined borders in 10/11 patients with PM from renal cancer.

As it is not possible to identify a typical endosonographic image for a PM, it seems reasonable to assume that the identification by EUS of a heterogeneous pancreatic mass with well-defined borders in a patient with a previous history of malignancy should firstly raise the suspicion for a PM, and EUS-FNA should be used to confirm the diagnosis. At this moment, we need to highlight the importance of the histopathologic diagnosis of the solid pancreatic lesions, which can avoid unnecessary surgery for non-neoplastic tumours, such as lymphoma, mycoses and tuberculosis, which were detected in almost 22% of our cases, all of them confirmed by EUS-FNA. Conversely, surgical resection can be indicated and offer a good survival for PM in selected patients, especially for renal tumours [27,37], which could be observed in 2 out of 4 patients with clear renal cell cancer submitted to surgery in our study, both of them still alive after 6 and 12 months. The use of histology was effective and made the correct diagnosis of PM in 94% of the cases in our experience.

Due to the absence of studies evaluating the histology of PM obtained by EUS-FNA, the authors believe this study has a considerable value. We found that EUS-FNA had a major clinical impact in 86% of patients with PM. It detected PM in patients whose CT only identified a pancreatic mass and labeled it as cancer, without raising the possibility of a PM. In addition, EUS-FNA detected the presence of PM prior to the identification of the new primary tumour in 6 (18%) cases, 5 of them confirmed by EUS after identification of a mass on CT, and another one in which CT demonstrated a subepithelial gastric tumour.

Unlike other studies which included patients only after a positive diagnosis of PM, we selected only cases with a previous treatment for an extrapancreatic cancer which, during their clinical follow-up, presented a pancreatic mass on CT, and were afterwards submitted to EUS-FNA [3,22]. Therefore, performance of histology obtained by EUS-FNA for diagnosis of PM could be assessed. Sensitivity, specificity, positive and negative predictive values, and accuracy of EUS-FNA with histology analysis of the specimens for diagnosis of PM were, respectively, 93.8%, 60%, 93.8%, 60% and 89%. Despite a good accuracy, we must point out that historical inaccuracies and biases are unavoidable in a retrospective database analysis, and the figures drawn from it must be taken cautiously. Two of our patients were found to have PM from previously unknown primary cancers. Besides, it is still possible that other patients with an unknown primary cancer may have undergone EUS-FNA for a pancreatic mass and may have a non-diagnostic histology sample. These patients would not be included and therefore will not appear as false negative cases. This raises the question about whether all PM has been included in the study, and could explain the low specificity and negative predictive values of the study.

As we know so far, there is no study evaluating the performance of the histology obtained by EUS-FNA for the diagnosis of PM. Fritscher-Ravens et al. [21] evaluated the role of the cytopathology for the diagnosis of PM in specimens obtained by EUS-FNA in a study with a small number of PM. Sensitivity, specificity, positive and negative predictive values, and accuracy were, respectively, 88%, 100%, 100%, 80%, and 92%. These results were somewhat better when compared with our figures. However, the endosonographer performing the procedure was trained for cytopathologic evaluation of the specimens. This way, despite the absence of an on-site cythopathologist, a better managing of the specimens could be offered in the moment of the exam. Besides, we need to remember that there was a very small number of patients, which is very important, because a larger number of cases with cytopathology revealing adenocarcinoma might constitute an important drawback for the differential diagnosis between a primary pancreatic malignancy and a PM, demanding more laborous techniques, such as imunocytochemistry and detection of specific tumoral markers. Moreover, the authors did not comment on the time required for each endoscopic procedure with cytophathologic evaluation. On the other hand, EUS-FNA with sampling for histology analysis without on-site cytopathologist neither required additional time for the endoscopic procedure, nor additional training for the endosonographer in regard to the management of the specimens. So, this approach with a very good sensitivity and accuracy for PM, could be more convenient and practical for the majority of the endosonographers working without on-site cytopathologists and expertise for the handling and evaluation of the aspirated material.

## Conclusions

This study demonstrates that PM should be considered in the presence of a solid pancreatic lesion in patients with a history of a previous extrapancreatic cancer, regardless of the time elapsed from the occurrence of the primary cancer. EUS-FNA with histology of the specimens is the best method for definitive diagnosis of pancreatic disease in this group of patients

## Abbreviations

EUS-FNA: Endoscopic ultrasound-guided fine-needle aspiration; PM: Pancreatic metastases; US: Ultrasound; CT: Computed tomography; IPMN: Intraductal papillary mucinous neoplasia; NET: Neuroendocrine tumour.

## Competing interests

The authors declare that they have no competing interests.

## Authors’ contributions

JCA was responsible for the conception and design of the study. CVL made substantive contributions to review the study critically for intellectual content, and final approval of the material to be published. RK participated in the acquisition, analysis and interpretation of data. FV carried out the acquisition, analysis and interpretation of pathology data. ERLF drafted and review the manuscript. JSS helped to review the manuscript for important intellectual content. All authors read and approved the final manuscript.

## Pre-publication history

The pre-publication history for this paper can be accessed here:

http://www.biomedcentral.com/1471-230X/13/63/prepub

## Supplementary Material

Additional file 1 **Clinical features, treatment and follow-up of patients with clinical history of extrapancreatic cancer submitted to EUS-FNA.** Symptoms - AP: abdominal pain; J: jaundice; WL: weight loss; AcP: acute pancreatitis. **Treatment** – CT: chemotherapy; RT: radiotherapy. **Surgery -** W: Whipple procedure; EL: exploratory laparotomy; SP: subtotal pancreatectomy. n/a: not applicable. Click here for file
