# Supplementary material for: Accuracy of endoscopic ultrasound-guided fine-needle aspiration in the suspicion of pancreatic metastases
Source: BMC Gastroenterol. 2013 Apr 11;13:63. doi: 10.1186/1471-230X-13-63 (PMC3651366; doi:10.1186/1471-230X-13-63)
Supplement: Additional file 1 — Clinical features, treatment and follow-up of patients with clinical history of extrapancreatic cancer submitted to EUS-FNA. Symptoms - AP: abdominal pain; J: jaundice; WL: weight loss; AcP: acute pancreatitis. Treatment – CT: chemotherapy; RT: radiotherapy. Surgery - W: Whipple procedure; EL: exploratory laparotomy; SP: subtotal pancreatectomy. n/a: not applicable. [file 1471-230X-13-63-S1.doc]

**Table 1**: Clinical features, treatment and follow-up of patients with clinical history of extrapancreatic cancer submitted to EUS-FNA

| **No** | **Age(y)/Gender** | **Primary Cancer** | **Symptoms** | **Recurrent Malignancy** | **CT findings** | **Diagnosis to recurrence (mo)** | **Size of the lesion (cm)** | **Diagnosis of EUS-FNA (micro-hystology)** | **Treatment** | **Surgery** | **Outcome** | **Survival after EUS (mo)** |
| --- | --- | --- | --- | --- | --- | --- | --- | --- | --- | --- | --- | --- |
| **1** | 63/M | Lung | AP | No | Mass | n/a | 4,9 | Squamous cell cancer | CT | No | Death | 9 |
| **2** | 48/M | Rhabdomyosarcoma | AP | Yes | Mass | 41 | 7,3 | Rhabdomyosarcoma | CT | No | Death | 17 |
| **3** | 57/M | Sarcoma | AP+WL | Yes | Mass | 23 | 3,8 | Leiomiosarcoma | CT | No | Death | 11 |
| **4** | 72/M | Colon | AP | Yes | Mass | 51 | 5,2 | Blastomicoses | Antifungal therapy | No | Alive | 89 |
| **5** | 58/M | Kidney | AP | Yes | Mass | 84 | 2,8 | Clear renal cell cancer | Surgery | SP | Death | 6 |
| **6** | 59/M | Lung | AP | Yes | Mass | 15 | 4,8 | Negative | Surgery | SP | Death | 9 |
| **7** | 42/M | Stomach | AP+WL | Yes | Mass | 23 | 5,2 | Negative | Surgery | W | Death | 6 |
| **8** | 65/F | Colon | AP | Yes | Mass | 37 | 2,5 | Adenocarcinoma | Surgery | EL | Death | 12 |
| **9** | 74/F | Colon | AP+WL | Yes | Mass | 25 | 1,2 | Adenocarcinoma | Surgery | W | Death | 13 |
| **10** | 78/F | Colon | AcP | Yes | Mass | 39 | 1,2 | Adenocarcinoma (signet ring cell) | None | No | Death | 5 |
| **11** | 57/M | Non-Hodgkin Lymphoma | AP | Yes | Mass | 12 | 5,4 | Lymphoma | CT | No | Death | 29 |
| **12** | 58/M | Skin | NONE | Yes | Mass | 36 | 3,4 | Tuberculosis | Antibacterial  therapy | No | Alive | 72 |
| **13** | 48/M | Skin | AP | Yes | Mass | 12 | 3,9 | Melanoma | None | No | Death | 11 |
| **14** | 66/F | Breast | NONE | Yes | Mass | 180 | 5,9 | Adenocarcinoma | None | No | Death | 7 |
| **15** | 62/M | Myeloma | NONE | Yes | Mass | 14 | 3,3 | Plasmocytoma | CT | No | Death | 15 |
| **16** | 32/M | Non-Hodgkin Lymphoma | AcP | Yes | Mass | 10 | 2,6 | Lymphoma | CT | No | Death | 24 |
| **17** | 78/M | Colon | NONE | Yes | Mass | 47 | 3,5 | Adenocarcinoma | Surgery | EL | Death | 14 |
| **18** | 71/M | Lung | NONE | No | Mass | n/a | 3,7 | Squamous cell cancer | CT/RT | No | Death | 13 |
| **19** | 34/M | Stomach | NONE | No | Mass | n/a | 7,8 | Adenocarcinoma (signet ring cell) | Surgery | EL | Death | 4 |
| **20** | 84/M | Kidney | J | No | Mass | n/a | 3,9 | Clear renal cell cancer | None | No | Death | 7 |
| **21** | 62/F | Ovarian | AP | No | GIST | n/a | 4,5 | Adenocarcinoma | CT | No | Death | 7 |
| **22** | 58/M | Non-Hodgkin Lymphoma | J | Yes | Normal pancreas | 6 | 1,4 | Lymphoma | CT | No | Death | 36 |
| **23** | 79/F | Non-Hodgkin Lymphoma | J | Yes | Increase of the pancreas | 8 | 2,3 | Lymphoma | CT | No | Death | 28 |
| **24** | 67/F | Breast | J | Yes | Increase of the pancreas | 60 | 3,9 | Adenocarcinoma | None | No | Death | 6 |
| **25** | 26/F | Liver | NONE | No | Mass | n/a | 3,7 | Hepatocellular carcinoma | CT | No | Death | 5 |
| **26** | 67/M | Esophageal | NONE | Yes | Mass | 37 | 4,2 | Squamous cell cancer | CT/RT | No | Death | 6 |
| **27** | 59/M | Mesothelioma | NONE | No | Mass | n/a | 7,5 | Mesothelioma | CT | No | Death | 9 |
| **28** | 81/F | Breast | NONE | Yes | Dilation of MPD | 60 | 1,9 | Adenocarcinoma | Surgery | W | Alive | 22 |
| **29** | 69/M | Gallbladder | AP | Yes | Mass | 36 | 2,5 | Adenocarcinoma | None | No | Death | 5 |
| **30** | 59/M | Leiomiossarcoma | J | Yes | Mass | 32 | 6,4 | Leiomiosarcoma | CT | No | Death | 6 |
| **31** | 66/M | Lung | AP | No | Mass | n/a | 3,9 | Small cell cancer | Surgery | W | Death | 8 |
| **32** | 58/M | Kidney | NONE | Yes | Mass | 59 | 2,2 | Clear renal cell cancer | Surgery | W | Alive | 12 |
| **33** | 70/M | Kidney | AP+WL | Yes | Mass | 120 | 5,6 | NET | Surgery | EL | Alive | 9 |
| **34** | 73/M | Non-Hodgkin Lymphoma | AP+WL | Yes | Mass | 2 | 12,7 | Lymphoma | CT | No | Death | 7 |
| **35** | 68/F | Bladder | NONE | Yes | Mass | 24 | 1,2 | IPMN | None | No | Death | 9 |
| **36** | 60/M | Non-Hodgkin Lymphoma | AP+WL | Yes | Mass | 2 | 7,2 | Lymphoma | CT | No | Death | 6 |
| **37** | 77/F | Kidney | NONE | Yes | Mass | 360 | 2,3 | Clear renal cell cancer | Surgery | SP | Alive | 6 |

**Symptoms -** AP: abdominal pain; J: jaundice; WL: weight loss; AcP: acute pancreatitis. **Treatment** – CT: chemotherapy; RT: radiotherapy. **Surgery -** W: Whipple procedure; EL: exploratory laparotomy; SP: subtotal pancreatectomy. n/a: not applicable.
